# The NK-1 Receptor Antagonist L-732,138 Induces Apoptosis and Counteracts Substance P-Related Mitogenesis in Human Melanoma Cell Lines

**DOI:** 10.3390/cancers2020611

**Published:** 2010-04-20

**Authors:** Miguel Muñoz, Marisa Rosso, Ana González-Ortega, Rafael Coveñas

**Affiliations:** 1 Research Laboratory on Neuropeptides, Virgen del Rocío University Hospital, Sevilla, Spain; E-Mails: marisarossog@gmail.com (M.R.); agonzalezbio@yahoo.com (A.G.-O.); 2 Institute of Neurosciences of Castilla y León (INCYL), Laboratory of Neuroanatomy of the Peptidergic Systems (Laboratory 14), Salamanca, Spain; E-Mail: covenas@usal.es (R.C.)

**Keywords:** melanoma, NK-1 receptor antagonist, L-732,138, antitumor, apoptosis

## Abstract

It has been recently demonstrated that substance P (SP) and neurokinin-1 (NK-1) receptor antagonists induce cell proliferation and cell inhibition in human melanoma cells, respectively. However, the antitumor action of the NK-1 receptor antagonist L-732,138 on such cells is unknown. The aim of this study was to demonstrate an antitumor action of L-732,138 against three human melanoma cell lines (COLO 858, MEL HO, COLO 679). We found that L-732,138 elicits cell growth inhibition in a concentration dependent manner in the melanoma cells studied. Moreover, L-732,138 blocks SP mitogen stimulation. The specific antitumor action of L-732,138 occurred through the NK-1 receptor and melanoma cell death was by apoptosis. These findings indicate that the NK-1 receptor antagonist L-732,138 could be a new antitumor agent in the treatment of human melanoma.

## 1. Introduction

Melanoma is a neoplasm originating from melanocytes, which are derived from the neural crest. It represents 1% of cancers and it accounts for approximately 65% of skin cancer deaths. Melanoma now accounts for approximately 4% of all cancers diagnosed in the United States [1]. Presently, primary cutaneous malignant melanoma can be effectively managed with surgical treatment, obtaining high survival rates after five years follow-up. However, survival dramatically decreases in stages III and IV of this tumor. In these stages, an effective treatment does not exist. The last two decades have seen no significant progress in extending the survival of patients with distant metastases, despite multiple trials of cytotoxic chemotherapy agents. Therefore, there is an urgent need to improve therapy in melanoma patients. 

Substance P (SP) is an undecapeptide that belongs to the tachykinin family of peptides. It is known that SP, neurokinin A (NKA), neuropeptide K and neuropeptide Gamma (the two latter elongated forms of NKA) are derived from the preprotachykinin A gene, whereas neurokinin B (NKB) is derived from the preprotachykinin B gene. The biological actions of SP, NKA and NKB are mediated by three receptors, named neurokinin (NK)-1, NK-2 and NK-3; the NK-1 receptor showing preferential affinity for SP. After binding to the NK-1 receptor, SP regulates many biological functions [2,3] and this neuropeptide has also been implicated in neurogenic inflammation, pain and depression [4] as well as in tumor cell proliferation, neoangiogenesis and metastasis [5,6]. In addition, the expression of SP in primary invasive malignant melanomas, metastatic melanomas, melanomas *in situ*, atypical (dysplastic) nevi, and spindle and epithelioid cell (Spitz) nevi has been described, but it was not detected in any acquired benign melanocytic nevi [7]. Moreover, it was recently reported that human malignant melanoma cell lines and melanoma samples express NK-1 receptors [8]. It has also been demonstrated that the activation of the NK-1 receptor by SP induces mitogenesis in several melanoma cell lines [9,10,11] and in other tumor cell lines [12,13,14,15,16,17,18,19,20]. In addition, it is known that SP is a main mediator of capillary vessel growth *in vivo* and of the proliferation of cultured endothelial cells *in vitro*, and that SP also induces neoangiogenesis [21]. Moreover, the active migration of tumor cells, a crucial requirement for invasion and metastasis development, is regulated by SP signals [22]. 

The NK-1 receptor antagonist L-733,060 (a piperidine derivative) showed a high affinity for the human NK-1 receptor *in vitro* [23]. We have also demonstrated that L-733,060 shows antitumor activity against human malignant melanoma cell lines [10] and against neuroblastoma, glioma, retinoblastoma, pancreas, larynx, gastric and colon carcinoma cell lines [10,15,16,19,20]. Furthermore, it has been recently reported that the NK-1 receptor antagonist aprepitant (a morpholine derivative) is a broad spectrum antitumor drug [24] and exerts an antitumor action against human malignant melanoma cell lines [8]. Additionally, the NK-1 receptor antagonist L-732,138 (an L-tryptophan derivative) showed a competitive and selective antagonism for the NK-1 receptor. It is approximately 1,000-fold more potent in cloned human NK-1 receptors than in cloned human NK-2 and NK-3 receptors; and approximately 200-fold more potent in human NK-1 receptors than in rat NK-1 receptors [25]. It is known that the administration of L-732,138 exerts an attenuation of hyperalgesia [26] and it has been also described that L-732,138 is able to antagonize H(3) antagonist-induced skin vascular permeability [27]. In addition, antitumor activity against glioma, neuroblastoma, retinoblastoma and larynx carcinoma was shown [17,18,19,28]. However, to our knowledge it is unknown whether the antitumor action of the NK-1 receptor antagonist L-732,138 is exerted, or not, on human malignant melanomas. Thus, the aims of this study are: (1) To demonstrate, using a MTS colorimetric method to evaluate cell viability, the antitumor action of the NK-1 receptor antagonist L-732,138 against human melanoma COLO 858, MEL HO, and COLO 679 cell lines, and to show that this antitumor action occurs through the NK-1 receptor; and (2) To investigate whether the NK-1 receptor antagonist L-732,138 produces apoptosis in the three melanoma cell lines studied. 

## 2. Material and Methods

### 2.1. Cell Culture

We used COLO 858 (ICLC Interlab Cell Line Collection-CBA-Genova), MEL HO and COLO 679 (DSMZ-Deutsche Sammlung von Mikroorganismen und Zellkulturen) human melanoma cell lines. These cell lines were incubated at 37 ºC in a humidified atmosphere of 95% air/5% CO_2_, according to the manufacturer’s instruction. 

### 2.2. Drug Treatments

The NK-1 receptor antagonist N-acetyl-L-tryptophan 3, 5-bis (trifluoromethyl) benzyl ester, molecular weight 472.39 (L-732,138) (Sigma-Aldrich, Madrid, Spain), was dissolved in distilled water containing 0.2% dimethylsulphoxide (DMSO) before sample treatment. In order to determine the IC_50_, different concentrations (10 to 100 µM) of L-732,138 were evaluated. SP, acetate salt (Sigma-Aldrich, Madrid, Spain), was dissolved in distilled water and different concentrations of SP (5, 10, 50, 100 and 500 nM) were used. The most mitogenic nanomolar SP concentration for each cell line was incubated 1 h before the addition of L-732,138.

### 2.3. Proliferation Assays

Cell proliferation was evaluated using the tetrazolium compound 3-(4, 5-dimethylthiazol-2-yl)-5-(3-carboxymethoxyphenyl)2-(4-sulfophenyl)-2H-tetrazolium, inner salt (MTS), according to the manufacturer’s instructions (CellTiter 96 Aqueous One Solution Cell Proliferation Assay, Promega Corp., Madison, USA). Cell numbers were quantified using a Coulter counter. The plate included blank wells (0 cells/0.1 mL), control wells (10^4^ cells/0.1 mL), control wells with DMSO, control wells treated with L-732,138 and control wells treated with the most effective SP concentration and L-732,138. The plates were inoculated with L-732,138 (10-100 µM for tumor cell lines) and were incubated for the first doubling time specific for each tumor cell line. Moreover, control wells were treated with different concentrations of SP. For the proliferation assay, 20 µL of the MTS reagent was added to each well 90 min before reading the samples on a multiscanner microplate reader (TECAN Spectra classic, Barcelona, Spain) at 492 nm. Each experimental condition was assayed in duplicate and all experiments were performed at least three times.

### 2.4. Statistical Analyses

Data were expressed as means ± SD. Statistical analysis was performed with SPPS statistical software for Microsoft Windows, release 14.0 (Professional Statistic, Chicago, IL). The homogeneity of the variance was tested using the Levene test. If the variances were homogeneous, the data were analyzed by using the one-way ANOVA test with Bonferroni’s correction for multiple comparisons. For data sets with non-homogeneous variances, the ANOVA test with T3 Dunnett posthoc analysis was applied. The criterion for significance was *P* < 0.05 for all comparisons. 

### 2.5. DAPI Staining

In order to determine whether apoptosis was induced by the NK-1 receptor antagonist L-732,138, DAPI staining was performed. Briefly, after treatment with L-732,138 for their first doubling time approximately, the cells were fixed in 4% paraformaldehyde. Following a second wash in PBS, cells were incubated in 1 μg/mL DAPI solution (Sigma-Aldrich) for 30 min in the dark. The cells were then observed under a fluorescence microscope (Zeiss, Oberkochen, Germany). Apoptotic cells were defined by the chromatin condensation and nuclear fragmentation. We counted the number of apoptotic cells. In each case, the count was repeated in three different slides. Finally, in each slide, we counted the number of apoptotic cells located in five different sequentially fields. 

## 3. Results

### 3.1. Antitumor Action of L-732,138

**Figure 1:**
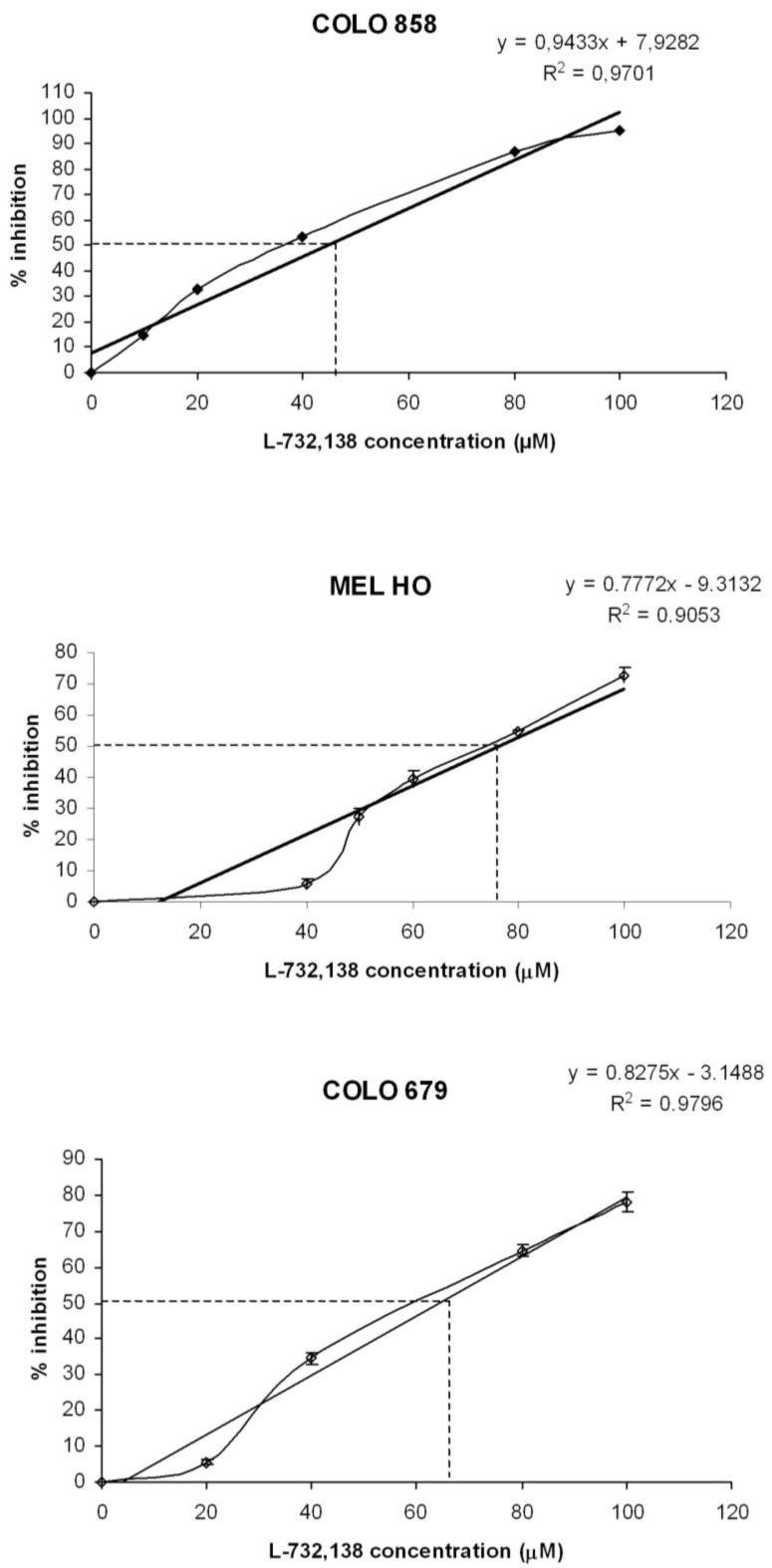
Percentage of growth inhibition of COLO 858, MEL HO and COLO 679 human melanoma cells at first doubling time in *in vitro* cultures following the addition of increasing concentrations (10 to 100 μM) of L-732,138. Level of significance: * *p* ≤ 0.05.

After the administration of increasing concentrations of L-732,138, we observed growth inhibition in all the melanoma cell lines studied (Figure 1). Moreover, treatment of the melanoma cell lines with L-732,138 resulted in a concentration-dependent cytotoxicity (see Figure 1). The IC_50_ and IC_100_ growth inhibition concentration of L-732,138 on melanoma cell lines are shown in Table 1. Maximum inhibition was observed when the drug was present at a concentration of 100 µM during the culture periods. At the first doubling time, a strong decrease in the number of cells in the cell lines studied here was found at intermediate concentrations and no living or very few cells were observed at the maximal concentration. A lower inhibition of growth of the melanoma cell lines was observed in the presence of low doses of L-732,138. There were no significant differences between the control wells and the control wells-DMSO (data not shown).

**Table 1:** Half inhibition (IC_50_) and maximum inhibition (IC_100_) of melanoma cell lines after administration of the NK-1 receptor antagonist L-732,138.

Melanoma cell line	L-732,138	
	IC_50_ μM	IC_100_ μM
COLO 858	44.6	97.6
MEL-H0	76.3	140.6
COLO 679	64.2	124.7

**Figure 2:**
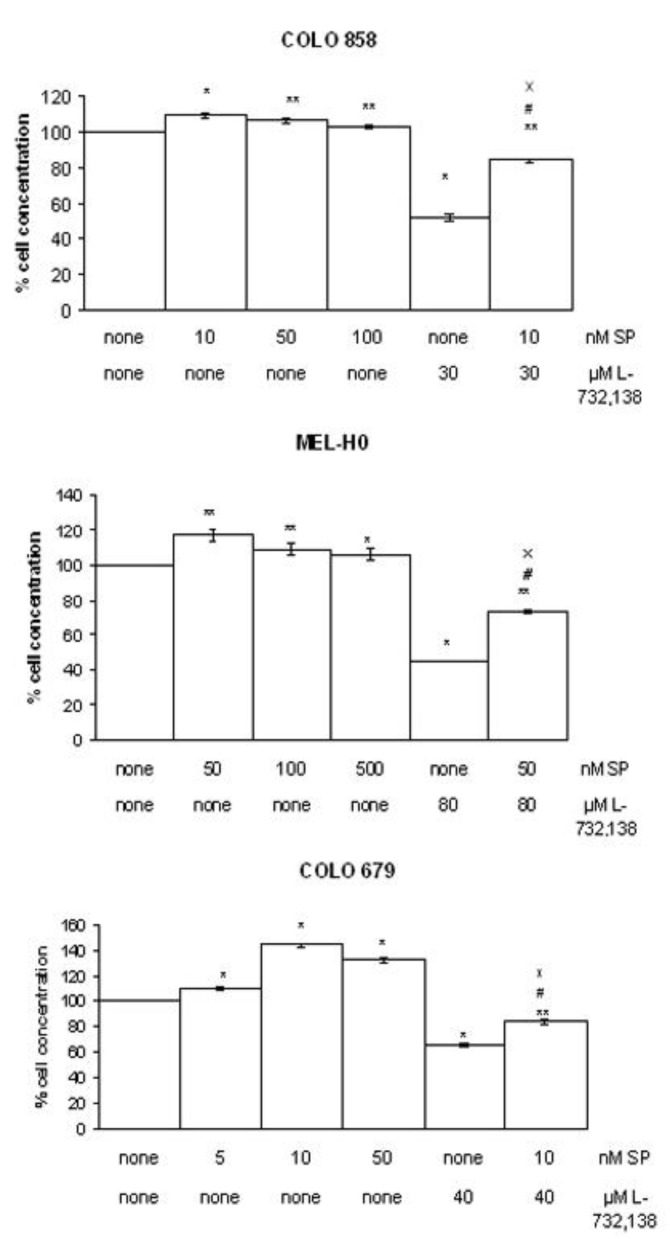
Induction of cell proliferation of COLO 858, MEL HO and COLO 679 human melanoma cells by SP at several nanomolar concentrations (5, 10, 50, 100 and 500 nM) according to the cell line studied. The NK-1 receptor antagonist L-732,138 was added in the presence (the most mitogenic exogenous SP nM concentration) or absence (none) of SP during the first doubling time. In both cases, L-732,138 inhibited cell proliferation. Using the ANOVA test, a significant difference between each group and the control group (none-none) was found. Level of significance: ** p ≤ 0.05 and # and x p ≤ 0.05. Vertical bars indicate SD.

After the administration of different doses of SP, our group has previously reported an increase in the growth of the three melanoma cell lines studied here [9]. In order to examine whether the NK-1 receptor antagonist L-732,138 inhibited melanoma cell proliferation via an interaction with its receptor, we used the specific NK-1 receptor agonist SP in competition experiments (Figure 2). Here, the cellular concentration at IC_50_ µM of L-732,138 and nM of SP was lower than that observed with different doses of SP alone for the melanoma cell lines studied here. These results indicate that L-732,138 blocks SP mitogen stimulation. Moreover, L-732,138-induced growth inhibition was partially reversed by the administration of the most mitogenic nanomolar dose of exogenous SP. These data indicate the specificity of the NK-1 receptor activation in the growth of the melanoma cell lines by SP, since an increase in the cellular concentration was observed in the cell lines studied with respect to the values found when the antagonist was administered alone (Figure 2). 

### 3.2. Apoptosis

**Figure 3:**
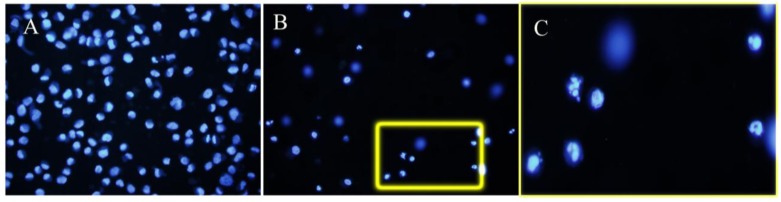
Culture of COLO 858 melanoma cells not treated (A) or treated with the NK-1 receptor antagonist L-732,138 (B). (C) High power magnification of the region delimited by the yellow rectangle in B. Note the apoptotic figures. Chromatin condensation and nuclear fragmentation are observed.

After administration of L-732,138, a large number of apoptotic cells were found in COLO 858, MEL HO and COLO 679 melanoma cell lines (Figure 3). In DAPI-stained cultures, at IC_50_ concentration we observed a mean of 43.6 ± 2.6 (SD)% apoptotic cells for the three melanoma cell lines, whereas at IC_100_ concentration we found 51.4 ± 4.5 (SD)% apoptotic cells. Conversely, in control melanoma cells (not treated with L-732,138) we observed a mean of 0.1 ± 0.2 (SD)% apoptotic cells for the three melanoma cell lines. 

## 4. Discussion

We have demonstrated for the first time the antitumor action of the NK-1 receptor antagonist L-732,138 in human melanoma COLO 858, MEL HO and COLO 679 cell lines. We have also demonstrated that the cell death observed in this study was due to a specific toxic effect of L-732,138 and not to a non-specific action of this drug. In our competition experiments, exogenous SP cell proliferation was partially reversed by the administration of L-732,138, suggesting the specificity of the NK-1 receptor blocking by L-732,138. Moreover, it has been reported that the above-mentioned melanoma cell lines express several isoforms of the NK-1 receptor [8]. Thus, the antitumor action on these melanoma cell lines by L-732,138 is probably related to the ability of this antagonist to block the NK-1 receptors expressed by the melanoma cell lines studied here. In addition, the findings of the present study demonstrate that treatment of COLO 858, MEL HO and COLO 679 cell lines with L-732,138 results in cell death and that such death occurs by apoptosis. This is in agreement with previous *in vitro* studies carried out in lung cancer [29], rhabdomyosarcoma [11], neuroblastoma, retinoblastoma, larynx, gastric and colon carcinoma cell lines [18,19,20,28]. It is also in agreement with previous studies reported by our group, in which the NK-1 receptor antagonists L-733,060/L-732,138 and the drug aprepitant exerted an antitumor action on glioma, neuroblastoma, melanoma, retinoblastoma, pancreas, larynx, gastric and colon carcinoma cell lines [9,10,15,16,17,18,19,20,24,28]. In addition, it has been recently demonstrated that aprepitant exerted an antitumoral action on human maligant melanomas [8]. Moreover, SP analogue antagonists (synonymous of NK-1 receptor antagonists) inhibited, both *in vitro* and *in vivo*, the growth of small cell lung cancer [3], as well as it is known that other NK-1 receptor antagonists inhibited *in vivo* the growth of glioma and breast carcinoma cells [30,31]. 

This suggests that NK-1 receptor antagonists block the NK-1 receptors expressed by tumor cells and that the structurally very different molecules piperidine (L-733,060), L-tryptophan (L-732,138) and morpholine (aprepitant) exert the same antitumor action (these molecules only have their specificity for the NK-1 receptor in common). In this sense, it seems that the antitumor action of the NK-1 receptor antagonists is related to stereochemical features and no to the chemical structure of the molecules. In sum, the data suggest the possibility of a common mechanism for cancer cell proliferation mediated by SP and NK-1 receptors. Were this the case, it would mean that NK-1 receptor antagonists L-732,138 could inhibit a large number of tumor cell types in which both SP and NK-1 receptors are expressed [18,20,32,33,34,35]. Moreover, it has been demonstrated that the NK-1 receptor expression is increased 25-36-fold in human pancreatic cancer cell lines in comparison with normal controls, and that tumors samples of patients with advanced tumor stages exhibit significantly higher NK-1 receptor levels [33]. Thus, NK-1 receptor visualization, after using immunohistochemical methods, will facilitate the identification of tumors with a sufficient NK-1 receptor overexpression for diagnostic and therapeutic intervention administrating NK-1 receptor antagonists [36]. In addition, it is known that melanoma tissues expressed NK-1 receptors, as well as human maligant melanomas [8]. Furthermore, it is known that the activation of the NK-1 receptor by SP, at nanomolar concentrations, induces mitogenesis in human malignant melanoma cell lines [9] and in other cancer cell lines [18,19,20,28], and that SP is expressed in primary invasive malignant melanomas and metastatic melanomas [37]. 

Moreover, it has been reported the effect of SP, a main mediator of neurogenic inflammation, on the growth of capillary vessels *in vivo*, and on the proliferation of cultured endothelial cells *in vitro*, as well as it has been demonstrated that NK-1 receptor agonists also induced neoangiogenesis probably through induction of endothelial cell proliferation, and this response is blocked specifically by NK-1 receptor antagonists [38]. Furthermore, neoangiogenesis is a sequential process, starting with early endothelial proliferation followed by new vessel formation and increased blood flow, then with maturation of endogenous neurovascular regulatory systems occurring later in this process in inflamed tissues [39,40]. Also, it has been associated with increased tissue innervations and expression of NK-1 receptors [34]. In the great majority of the investigate tumors, NK-1 receptors were found on intra- and peritumoral blood vessels [32]. In fact, NK-1 receptors were observed in smooth muscle cells of the small- and medium-caliber blood vessels, which were located in the peritumoral area and, occasionally such receptors were also observed in the endothelial cells of these vessels in the melanoma samples [8]. Additionally, one of the known functions of SP is to mediate the release of histamine from mast cells, frequently concentrate in the connective tissue surrounding many tumors including melanomas, and do so prior to the development of neoangiogenesis. By contrary, SP analogue antagonists (synonymous of the NK-1 receptor antagonists) inhibit tumor growth in pancreatic cancer *via* a dual mechanism that involves both antiproliferative and antiangiogenic properties [41]. Moreover, the active migration of tumor cells is a crucial requirement for metastasis development and cancer progression. It is known that SP induces the migration of tumor cells to specific organs by binding to the NK-1 receptor in cancer cells, where it can be blocked by NK-1 receptor antagonists [22]. These data suggest that SP and NK-1 receptors could play an important role in the development of invasion and metastasis. Additionally, some alarming signs of the malignization of nevi are: pain, pruritus, inflammation and increased size. These signs could be due to an increase of SP in the perilesional zone of the skin, produced by the different mechanisms involved in the neurogenic inflammation. In fact, the release of SP from the peripheral sensory fibres could produce vasodilatation, increases in vascular permeability, plasma extravasation and the release of histamine (pruritus) from skin mast cells. In addition, melanoma cells releasing SP could produce tumor proliferation.

All the data mentioned above suggest that the treatment of melanoma cell lines (expressing NK-1 receptors) with the NK-1 receptor antagonist L-732,138 could improve melanoma treatment, because it exerts an antitumor action through three mechanisms: (1) An antiproliferative effect due to the inhibition of tumor cell growth, inducing cell death by apoptosis; (2) An inhibition of angiogenesis in the tumor mass; and (3) An inhibition of the migration of tumor cells (invasion and metastasis). Moreover, it has been reported that a NK-1 receptor antagonist (the drug aprepitant) was used in a placebo-controlled trial in patients with depression, showing that the safety and the tolerability of this drug were generally similar to placebo [42]. Recently, we have also reported the safety of aprepitant against human fibroblast cells; thus, the IC_50_ for fibroblast cells was approximately three times more than the IC_50_ of the tumor cells [24]. Moreover, it is known that the NK-1 receptor antagonists in the host present/display other beneficial effects such as: anti-inflammatory [43], analgesic [44], anxyolitic [45], antidepressant [42], antiemetic [46], hepatoprotector [43], and neuroprotector [47]. 

## 5. Conclusions

We describe for the first time the antitumor action of the NK-1 receptor antagonist L-732,138 on MEL HO, COLO 858 and COLO 679 human malignant melanoma cell lines. Also, we have demonstrated that the antitumor action of L-732,138 is through the NK-1 receptor, and finally, that L-732,138 induces apoptosis in melanoma cell lines. All these observations suggest that the NK-1 receptor antagonist L-732,138 could be a novel and promising antitumor agent in the treatment of human melanoma. 
